# Eya2 Is Overexpressed in Human Prostate Cancer and Regulates Docetaxel Sensitivity and Mitochondrial Membrane Potential through AKT/Bcl-2 Signaling

**DOI:** 10.1155/2019/3808432

**Published:** 2019-06-16

**Authors:** Zhongyuan Liu, Long Zhao, Yongsheng Song

**Affiliations:** ^1^Department of Urology, Shengjing Hospital of China Medical University, China; ^2^Department of Nuclear Medicine, Shenzhen Hospital, Southern Medical University, China

## Abstract

The aberrant expression of Eya2 has been observed in a wide range of cancer types. However, the clinical significance and biological effects of EYA2 in human prostate cancer remain unknown. In this study, we showed that increased levels of Eya2 protein correlated with advanced TNM stage, T stage, and a higher Gleason score. Data from the Cancer Genome Atlas (TCGA) prostate cohort consistently revealed that Eya2 mRNA was positively correlated with a higher Gleason score, higher T stage, and positive nodal metastasis in prostate cancer. Furthermore, data from the Oncomine database showed increased levels of EYA2 mRNA expression in prostate cancer tissues compared with normal tissues. Eya2 protein expression was also higher in prostate cancer cell lines compared with a normal RWPE-1 cell line. We selected LNCaP and PC-3 cell lines for plasmid overexpression and shRNA knockdown. CCK-8, colony formation, and Matrigel invasion assays demonstrated that the overexpression of Eya2 promoted proliferation, colony number, and invasion while Eya2 shRNA inhibited proliferation rate, colony formation, and invasion ability. CCK-8 and Annexin V assays showed that Eya2 reduced sensitivity to docetaxel and docetaxel-induced apoptosis while Eya2 shRNA showed the opposite effects. The overexpression of Eya2 also downregulated the cleavage of caspase3 and PARP while Eya2 depletion upregulated caspase3 and PARP cleavage. Notably, JC-1 staining demonstrated that Eya2 upregulated mitochondrial membrane potential. We further revealed that the overexpression of Eya2 upregulated Bcl-2, matrix metalloproteinase 7 (MMP7), and AKT phosphorylation. Accordingly, data from the TCGA prostate cohort indicated that EYA2 mRNA was positively correlated with the expression of Bcl-2 and MMP7. The inhibition of AKT attenuated EYA2-induced Bcl-2 upregulation. In conclusion, our data demonstrated that Eya2 was upregulated in prostate cancers. EYA2 promotes cell proliferation and invasion as well as cancer progression by regulating docetaxel sensitivity and mitochondrial membrane potential, possibly via the AKT/Bcl-2 axis.

## 1. Introduction

Prostate cancer is the most frequently diagnosed cancer in men and is the second or third highest cause of death caused by cancer worldwide [1]. Despite improved therapies, the rate of recurrence for prostate cancer within five years remains approximately 25% [2, 3] and invasion is one of the main factors responsible for lethal consequences [4]. Docetaxel, which is currently used as the first-line therapy for patients with hormone-refractory prostate cancer (HRPC), is a taxane antimitotic agent and results in an overall improvement in survival. Acquired resistance to docetaxel can precede mortality in patients with HRPC and has also been shown to contribute to alterations in the invasive and motile phenotype of cells [5, 6]. Consequently, elucidating the mechanisms underlying prostate cancer invasion and chemoresistance is of great importance.

Eyes absent homolog 2 (Eya2) belongs to the eyes absent (EYA) family proteins which contain a highly conserved Eya domain and function as transcriptional cofactors with SIX family proteins. Over recent years, several studies have reported that Eya2 is involved in the progression of cancer. For example, Eya2 is known to be upregulated in human ovarian cancer and associated with poor survival in advanced cases of ovarian cancer [7]. Eya2 is reported to cooperate with Six1 to promote metastasis via the induction of TGF-*β* and epithelial-mesenchymal transition (EMT) in breast cancer cells [8]. Eya2 also facilitates astrocytoma invasion [9]. Other studies have shown that Eya2 is critical for PLZF-RARA-induced leukemogenesis [10]. Eya2 also promotes the proliferation of lung cancer cells by downregulating PTEN [11]. Collectively, these lines of evidence imply that Eya2 is a potential oncogene. However, the clinical significance and biological role of Eya2 in human prostate cancer remain unknown.

In the present study, we evaluated the expression pattern and biological characteristics of EYA2 in prostate cancer. Furthermore, we explored the potential molecular mechanisms underlying the chemosensitivity and mitochondrial function of EYA2 in prostate cancer cells.

## 2. Materials and Methods

### 2.1. Human Prostate Tissue

129 cases of human prostate tissue with the informed consent were obtained from patients treated in Shengjing Hospital of China Medical University between 2013 and 2016. The study was approved by the ethics review board of China Medical University. The sections were stained with hematoxylin and eosin stain for pathology diagnosis.

### 2.2. Immunohistochemistry

Formalin-fixed, paraffin-embedded tissues were used for immunohistochemistry staining. Generally, the sections were deparaffinized and rehydrated, and then the sections were boiled (2 min in 0.01 M citrate buffer pH 6.0) for antigen retrieval. After quenching of endogenous peroxidase activity with 0.3% H2O2 for 10 min and blocking with BSA for 30 min, sections were incubated at 4°C overnight with antibodies against Eya2 antibody (1:90 dilution rate, Sigma) using the Elivision Plus kit (MaiXin, Fuzhou, China). We used a scoring system including both staining intensity and percentage [12]. In each sample, five views were selected for evaluation. Nuclear localization was interpreted as positive staining. Staining intensity was scored as 0 (no/weak staining), 1 (moderate staining), and 2 (strong staining). The percentage of Eya2 nuclear staining was classified into 1:1% to25%, 2: 26%to50%, 3: 51%–75%, and 4: 76%-100%. These scores were multiplied to the final score. Cases with Eya2 final score <4 were considered as Eya2 low expression, and those with final score ≥4 were considered as Eya2 high expression (overexpression).

### 2.3. Cell Culture and Transfection

Prostate cancer cell lines including DU145, LNCaP, and PC-3 and normal cell line RWPE-1 were obtained from Shanghai Cell Bank, Chinese Academy of Sciences. Cells were cultured in PRMI-1640 with 10% fetal bovine serum (FBS). pcDNA 3.1 Eya2 plasmid was obtained from Addgene. Plasmid was transfected with Lipofectamine 3000.

shRNA sequences for Eya2 and scramble shRNA were cloned into lentiviral vector pZIP-hEF1-alpha-ZsGreen-Puro. The sh-Eya2 sequence was CGTGCGCATTGGCCTTATGAT and scramble shRNA sequence was GGAATCTCATTCGATGCATAC. Lentiviruses were produced by transfection of 293T cells using pMD2.G and pspax2 by Lipofectamine 3000. Lentiviral supernatant was collected at 48 hours, which was concentrated and used to infect target cells. Infected cells were selected with puromycin for one week.

### 2.4. Western Blot

For western blot, 60ug protein was added to SDS-PAGE, which was transferred to PVDF membranes (Millipore, MA, USA). Then, the membranes were incubated with the following primary antibodies at 4°C overnight: Eya2 (1:1000; Sigma, USA), MMP7, Bxl-2, AKT, p-AKT, caspase3, cleaved-caspase3, PARP and cleaved-PARP (1:1000; Cell Signaling Technology, USA), and GAPDH (1:2000; Cell Signaling Technology, USA). This was followed by secondary antibody incubation with anti-mouse or anti-rabbit horseradish peroxidase-conjugated secondary antibody (1:2000, Santa Cruz), and imaging on the imaging system (DNR, Isereal).

### 2.5. Realtime PCR

Cellular RNA was isolated using TRIZOL (Life technology). For quantitative PCR, cDNA was synthesized using the iScriptTM Reverse Transcription Supermix (Bio-Rad). The samples were then analyzed using SYBRGreen MasterMix on an ABI 7500 Realtime PCR system. The cycling condition was as follows: 50°C for 2min, 95°C for 2 min, 45 cycles of 95°C for 15 sec, and 60°C for 40 sec. *β*-actin was used as the endogenous calibrator control. Fold change of target gene was calculated according to 2^−ΔΔCt^ method.

### 2.6. CCK-8 and Colony Formation

*CCK-8 Assay.* Cell viability was assessed with the CCK-8 (Dojindo, Kumamoto, Japan). Cells were seeded into 96-well plates at about 3000 cells per well. 10 *μ*l CCK-8 reagent was added into each well. After incubation at 37°C for additional 2 h, cell proliferation was determined by examining the absorbance at a wavelength of 450 nm using a microplate reader (Bio-Rad, USA). Experiments were replicated in three times. Colony formation was as follows: cells (3000 per dish) were plated in 6cm dishes, cultured for about 2 weeks, and then stained with Giemsa.

### 2.7. Analysis of Annexin V/PI Staining and Mitochondrial Membrane Potential

Annexin V/PI kit (BD bioscience, USA) was used for analysis of apoptosis. The assays were performed on ACEA Flow and the data was analyzed by NovoExpress software.

Analysis of mitochondrial membrane potential was carried out using JC-1 staining kit (Life Technology). Cultured cells were incubated with staining buffer at 5 *μ*M concentration at 37°C for 30 minutes. Then, stained cells were washed with PBS and analyzed using a flow cytometer.

### 2.8. Statistical Analyses

We carried out statistical analysis using SPSS 17 software. We obtained data of Eya2 mRNA expression (gene chip) from Oncomine, which contains 52 prostate cancers with 50 normal prostate tissues. Eya2 RNA-seq data containing 497 cases of prostate cancers was obtained from The Cancer Genome Atlas (TCGA). Mann-Whitney U test was used to compare Eya2 mRNA in normal/cancerous tissues and cancers with/without nodal metastasis. Kruskal-Wallis H test was used to compare Eya2 mRNA in cancers with different Gleason scores and T stages. A *χ*2 test was used to examine possible correlations between Eya2 expression and clinicopathologic factors. Student's t-test was used to compare data obtained from biological experiments.* p*<0.05 was regarded as statistical significance.

## 3. Results

### 3.1. Eya2 Is Overexpressed in Prostate Cancers

**Figure 1:**
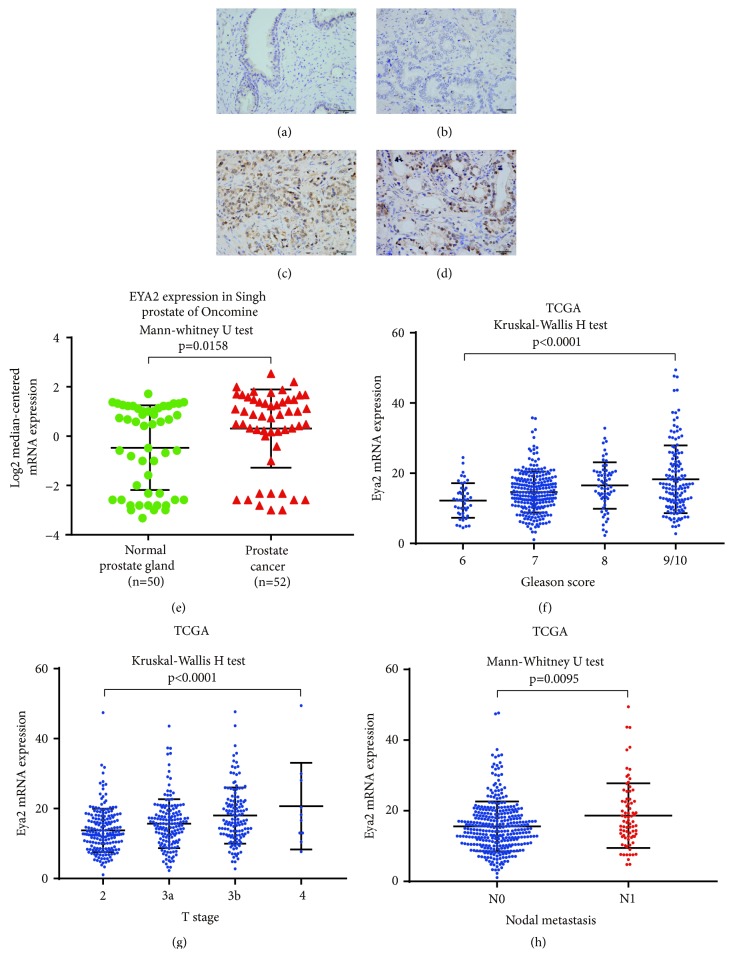
*Eya2 expression in prostate cancers.* (a) Negative or weak immunostaining of Eya2 in normal prostate tissue. (b) Negative Eya2 staining in prostate cancer. (c) Moderate nuclear Eya2 staining in a prostate cancer. (d) Strong nuclear Eya2 immunostaining in a case of prostate cancer. (e) Analysis of the Singh Prostate dataset of Oncomine. Eya2 mRNA was significantly elevated in prostate cancers compared with normal prostate tissues. (f) Eya2 mRNA in prostate cancers (TCGA). Eya2 mRNA positively correlated with Gleason score. (g) Eya2 mRNA was positively correlated with T stage (TCGA dataset). (h) Eya2 mRNA expression was significantly higher in prostate cancers with nodal metastasis (TCGA dataset). Statistical methods and p values are indicated in the figure.

The expression of Eya2 was analyzed in 98 prostate cancer tissues and 16 normal prostate tissues by immunohistochemistry. Normal prostate tissues showed negative or weak nuclear staining of Eya2 (Figure 1(a)) while prostate cancer specimens showed increased levels of Eya2 nuclear staining in 66 out of 98 (67.3%) cases (Figures 1(b)–1(d)). As shown in Table 1, high levels of Eya2 expression were positively correlated with a higher Gleason score (p=0.0128), advanced TNM stage (p=0.0038), and T stage (p=0.0008).

We analyzed gene chip data from Oncomine. The Singh Prostate dataset, provided by Oncomine, suggested that Eya2 was significantly elevated in prostate cancers (n=52) compared with normal prostate gland tissue (n=50) (Mann-Whitney U test, p=0.0158, Figure 1(e)). In addition, the Cancer Genome Atlas (TCGA) prostate cohort revealed that expression of Eya2 was significantly higher in prostate cancers with higher Gleason scores (Kruskal-Wallis H test, p<0.0001, Figure 1(f)), higher T stage (Kruskal-Wallis H test, p<0.0001, Figure 1(g)), and positive nodal metastasis (Mann-Whitney U test, p=0.0095, Figure 1(h))(Figures 1(f)–1(h)). Collectively, these data indicated that Eya2 is upregulated in human prostate cancers and correlates with malignant features.

### 3.2. Eya2 Promotes Proliferation and Invasion

**Figure 2:**
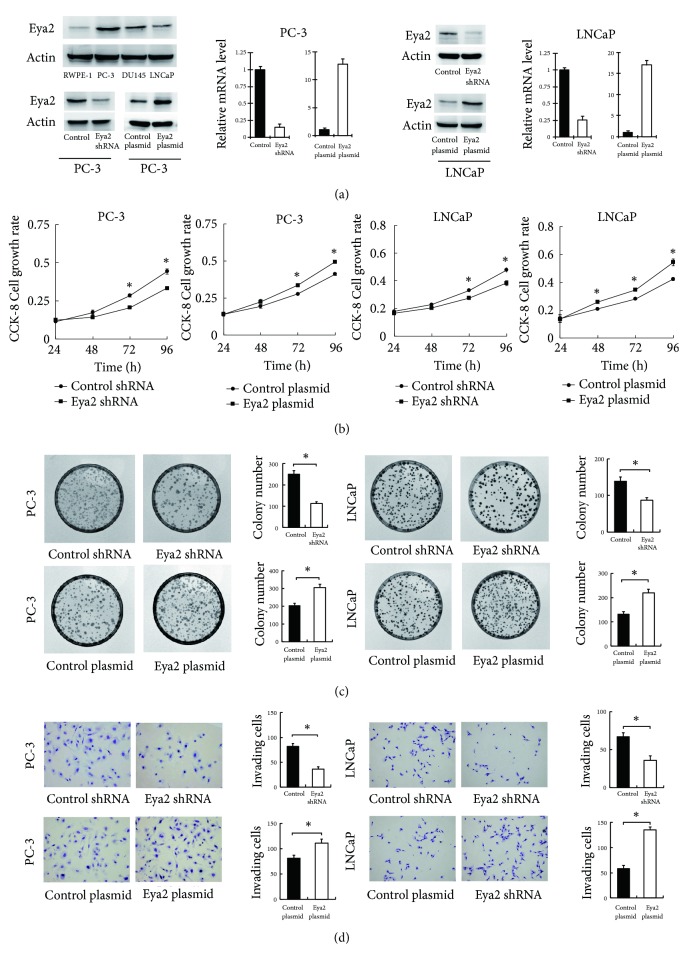
*Eya2 regulates the proliferation and invasion of prostate cancer.* (a) Western blot analysis of Eya2 protein in prostate cancer cell lines (LNCaP, PC-3, and DU145) and RWPE-1 (normal prostate cell line): there were higher levels of Eya2 protein in cancer cell lines than the normal cell line. Eya2 transfection significantly increased the levels of Eya2 protein and mRNA levels in PC-3 and LNCaP cells. The application of Eya2 shRNA significantly reduced the levels of Eya2 protein and mRNA levels in PC-3 and LNCaP cells. (b) Eya2 transfection led to an increase in proliferation rate in both PC-3 and LNCaP cell lines, while Eya2 shRNA knockdown reduced the proliferation rate. (c) Colony formation demonstrated that the overexpression of Eya2 upregulated colony number in both PC-3 and LNCaP cell lines, while Eya2 shRNA knockdown downregulated colony number. (d) Matrigel invasion assays demonstrated that the overexpression of Eya2 in both PC-3 and LNCaP cell lines increased the number of invading cells, while Eya2 shRNA knockdown decreased invading cell numbers. *∗* p<0.05.

Eya2 protein expression was examined in the normal prostate cell line RWPE-1 and three prostate cancer cell lines (PC-3, LNCaP, and DU145). The expression of Eya2 was higher in prostate cancer cell lines compared with the normal RWPE-1 cell line (Figure 1(a)). We transfected both LNCaP and PC-3 cell lines with the Eya2 plasmid and Eya2 shRNA. The efficiency of transfection was confirmed at both the mRNA and protein levels by RT-qPCR and Western blotting. We also performed CCK-8 assays and found that the depletion of Eya2 impaired proliferation while the transfection of Eya2 increased the rate of proliferation in both cell lines (Figure 2(b)). In accordance, colony formation assays revealed that the ectopic expression of Eya2 enhanced colony numbers while shRNA treatment reduced colony numbers (Figure 2(c)). Moreover, Matrigel invasion assays showed that Eya2 transfection increased the invasion ability of prostate cancer cells while shRNA decreased this ability (Figure 2(d)).

### 3.3. Eya2 Reduces Docetaxel Sensitivity and Downregulates Caspase 3 Cleavage

**Figure 3:**
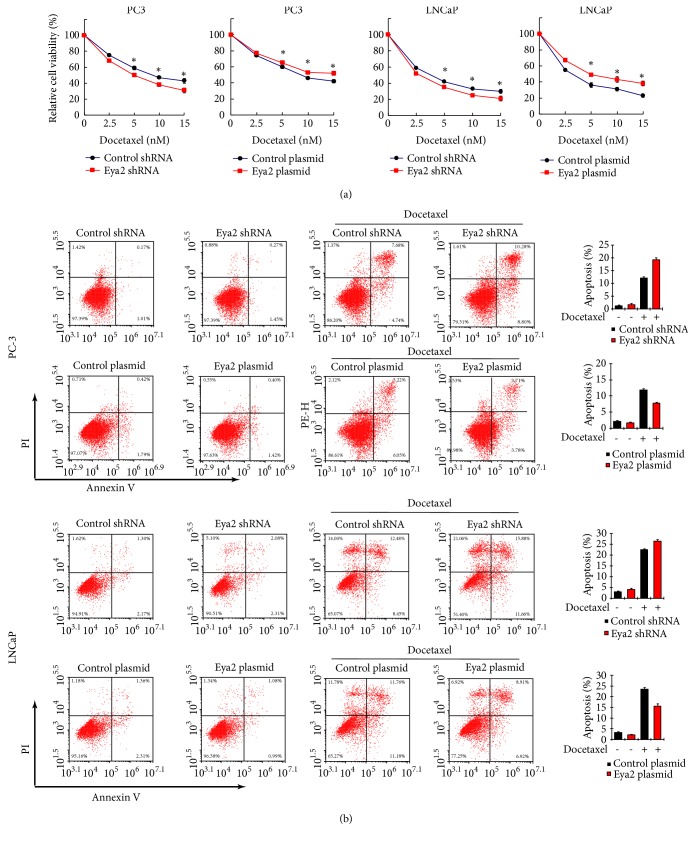
*Eya2 regulates docetaxel sensitivity and apoptosis.* (a) PC-3 and LNCaP cells experiencing Eya2 transfection/shRNA knockdown were treated with different concentration of docetaxel (2.5, 5, 10, and 15 nM). CCK-8 assays were used to determine cell viability. The overexpression of Eya2 led to an increase in cell viability in both cell lines while the depletion of Eya2 led to downregulated cell viability. (b) PC-3 and LNCaP cells experiencing Eya2 transfection/shRNA knockdown were treated with 5nM docetaxel for 24 h. Annexin V/PI staining showed that Eya2 overexpression inhibited the docetaxel-induced rate of apoptosis. Eya2 shRNA increased the rate of docetaxel-induced apoptosis. *∗ p* < 0.05.

**Figure 4:**
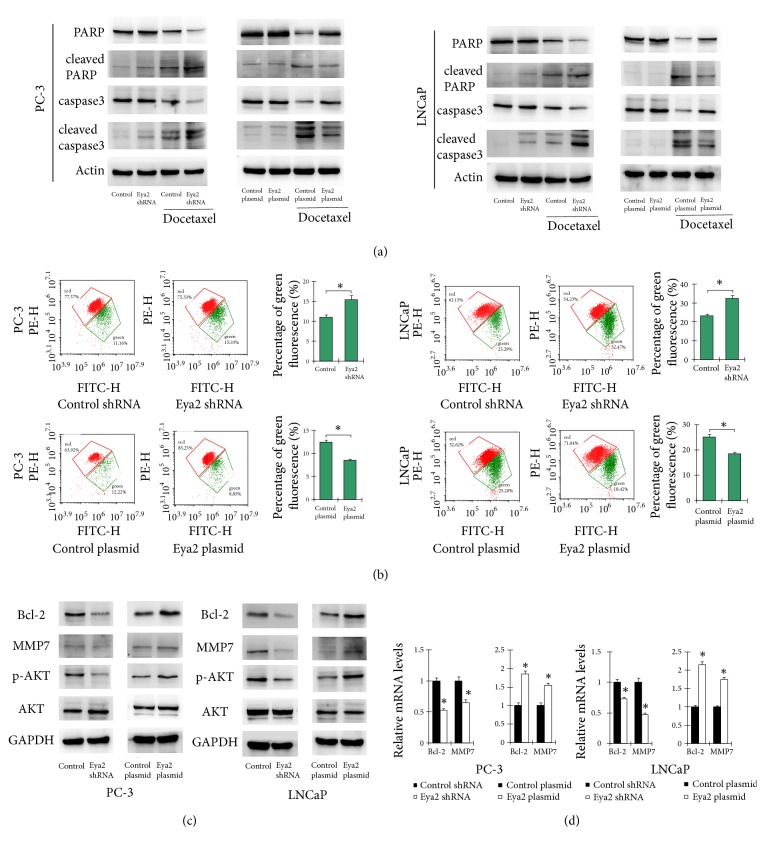
*Eya2 regulates mitochondrial membrane potential and related proteins.* (a) The overexpression of Eya2 inhibited the expression of cleaved-caspase3, cleaved-PARP and upregulated caspase3, PARP in docetaxel treated cells. The knockdown of Eya2 using shRNA led to an upregulation in the levels of cleaved-caspase3, cleaved-PARP and downregulated caspase3, PARP in docetaxel treated cells. (b) JC-1 staining was used to determine Δ*ψ*m. JC-1 is normally visualized as green when Δ*ψ*m is reduced. Our JC-1 staining results showed that the overexpression of Eya2 upregulated the Δ*ψ*m (as shown by a reduction in the proportion of green staining) while Eya2 shRNA reduced Δ*ψ*m (as shown by an increased proportion of green staining) after CDDP treatment. (c) Protein levels of Bcl-2, MMP7, p-AKT, and AKT in prostate cancer cells transfected with the Eya2 plasmid and shRNA; the overexpression of Eya2 increased the expression of Bcl-2, MMP7, and p-AKT proteins while the application of shRNA caused levels of these proteins to decrease. (d) Realtime PCR showed that the overexpression of Eya2 o increased the mRNA levels of Bcl-2 and MMP7 in prostate cancer cells while the application of shRNA caused a reduction in the expression of these proteins. *∗* p<0.05.

Next, we assessed whether Eya2 attenuated the sensitivity of prostate cancer cells to docetaxel. Cells in which Eya2 was overexpressed, or knocked down, were treated with different concentrations of docetaxel for 24 h. CCK-8 assays were then carried out to determine cell viability. As shown in Figure 3(a), the overexpression of Eya2 in PC-3 and LNCap cells reduced docetaxel sensitivity while the depletion of Eya2 enhanced docetaxel sensitivity. AnnxinV/PI staining demonstrated that ectopic Eya2 expression was greatly reduced while Eya2 depletion enhanced the rate of apoptosis in cells treated with docetaxel (Figure 3(b)). We also determined changes in the expression of caspase 3 and PARP. As shown in Figure 4(a), following docetaxel treatment, Eya2 shRNA led to an increase in caspase 3 and cleaved PARP levels and a reduction in the level of total caspase 3. The overexpression of Eya2 inhibited the cleavage of caspase 3 and PARP. These results indicated that Eya2 attenuates docetaxel sensitivity and facilitates drug resistance in prostate cancer cells.

### 3.4. Eya2 Regulates Mitochondrial Membrane Potential

Mitochondrial function is often closely associated with drug resistance. We therefore evaluated whether Eya2 could regulate mitochondrial membrane potential (Δ*ψ*m). In normal cells with high Δ*ψ*M, JC-1 is known to form complexes known as J-aggregates which exhibit intense red fluorescence. However, in cells with low Δ*ψ*M, JC-1 remains in the monomeric form and exhibits green fluorescence. The higher the ratio of red to green fluorescence, the higher the level of polarization in the mitochondrial membrane. As shown in Figure 4(b), in both PC-3 and LNCaP cells treated with docetaxel, the overexpression of Eya2 reduced the proportion of green staining. Our data showed that Eya2 enhanced Δ*ψ*m; Eya2 shRNA exhibited the opposite effect.

### 3.5. Eya2 Regulates Bcl-2 and MMP7 Expression

**Figure 5:**
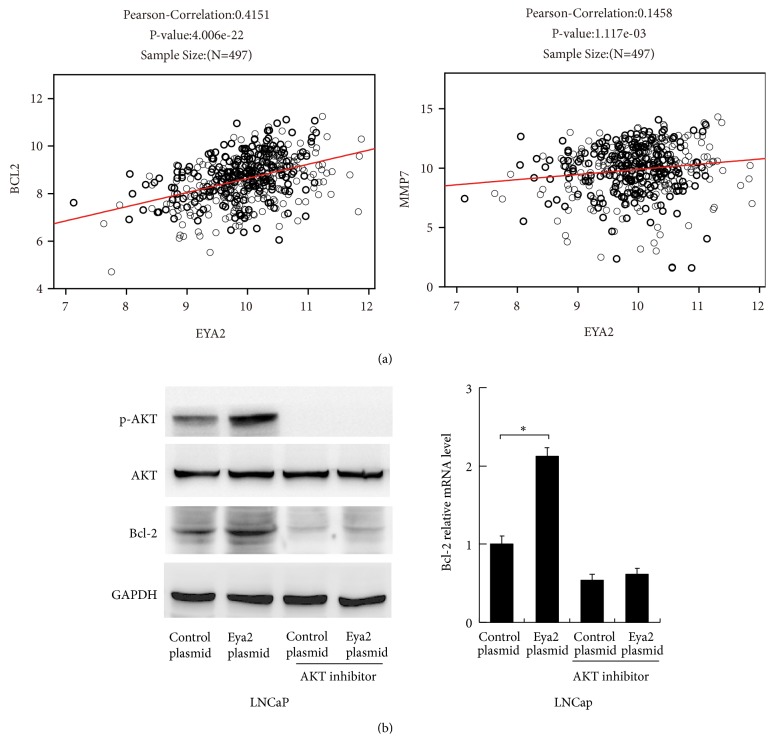
*Eya2 upregulates Bcl-2 via the AKT signaling pathway.* (a) We identified positive associations between Eya2 mRNA and Bcl-2/MMP7 mRNA in 497 cases of prostate cancer (Pearson's correlation analysis, RNA-seq data was obtained from the TCGA database). (b) Protein levels of Bcl-2, p-AKT, and AKT in LNCaP cells treated with Eya2 and the AKT inhibitor, perifosine. Realtime PCR analysis of Bcl-2 mRNA in LNCaP cells treated with Eya2 plasmid and the AKT inhibitor, perifosine. Eya2 overexpression failed to upregulate levels of Bcl-2 mRNA and protein in cells treated with the AKT inhibitor.

To identify the Eya2-associated mechanisms associated with apoptosis and invasion, we screened a range of potentially-related genes. Our results revealed that Eya2 could upregulate the expression of Bcl-2 and matrix metalloproteinase 7 (MMP7) at both the mRNA and protein level (Figures 4(c) and 4(d)). Consistent with these findings, 497 cases from the TCGA prostate cohort showed remarkably strong positive correlations between Eya2 and Bcl-2v (Pearson's correlation: 0.4151, p<0.01) and MMP7 (Pearson's correlation: 0.1458, p<0.01) at the mRNA level (Figure 5(a)).

### 3.6. Eya2 Regulates Bcl-2 via AKT Signaling Pathway

Next, we explored the potential mechanisms responsible for how Eya regulates the levels of Bcl-2. Since Bcl-2 is a downstream target of the AKT pathway, we first investigated whether the AKT pathway was involved. As shown in Figure 4(c), the overexpression of Eya2 upregulated p-AKT levels while the depletion of Eya2 downregulated p-AKT levels. We treated LNCaP cells with the AKT inhibitor perifosine (1*μ*M, 24h) and then tested the effect of Eya2 overexpression. As shown in Figure 5(b), the inhibition of AKT significantly downregulated Bcl-2 expression at both the mRNA and protein levels. In cells treated with AKT inhibitor, there was amelioration of Eya2-induced Bcl-2 expression, implying that the effect of Eya2 upon Bcl-2 was dependent upon AKT signaling, at least in part.

## 4. Discussion

An increasing body of evidence demonstrates that Eya2 functions as a cancer related protein in a range of human cancers including ovarian cancer, breast cancer, astrocytoma, and lung cancer [7–9, 11]. However, the clinical significance and biological functions of Eya2 in human prostate cancers remain to be fully elucidated. In the present study, we demonstrated that Eya2 was upregulated in both prostate cancers and cell lines. High levels of Eya2 expression were positively associated with Gleason scores and TNM stage. In addition, our results were supported by RNA-seq and gene chip data from TCGA and Oncomine. Collectively, our results highlight the fact that Eya2 is a potential biomarker for prostate cancer.

CCK-8 and colony formation assays indicated that the function of Eya2 is to promote cell proliferation. Matrigel invasion assays further showed that Eya2 promotes the invasion of prostate cancer, which was in accordance with the TCGA data showing positive correlations between Eya2 mRNA and T stage (invading depth) and nodal metastasis. We further demonstrated that Eya2 could upregulate the levels of MMP7 mRNA and protein. MMP7 is required to mediate cancer cell invasion [13] and has been reported to promote prostate cancer progression through induction of epithelial-to-mesenchymal transition (EMT) [14]. TCGA data further revealed a positive association between Eya2 and MMP7 mRNA. Collectively, these data suggested that Eya2 functions as a promoter for prostate cancer invasion, possibly by regulating MMP7.

The effect of Eya2 upon chemosensitivity has not yet been investigated. Our current findings are the first to reveal the role of Eya2 as a negative regulator of chemosensitivity. Chemotherapeutic drugs, such as docetaxel, induce apoptosis in a manner which is largely via a mitochondrial dependent pathway and changes in mitochondrial membrane potential often influence drug-induced apoptosis [15, 16]. Our results showed that Eya2 reduced docetaxel-induced apoptosis, prevented the downregulation of membrane potential, and inhibited the cleavage of caspase 3 and PARP. After screening several regulatory proteins, we found that Eya2 could upregulate Bcl-2, an important regulator of mitochondrial homeostasis [17, 18]. The protective role of Bcl-2 against apoptosis has been extensively reported in prostate cancers [19–21]. We also observed a positive correlation between Eya2 and Bcl-2 mRNA in the TCGA dataset. Collectively, our results indicate a link between Eya2 and the inhibition of apoptosis via Bcl-2 and mitochondrial function.

To further explore the possible mechanisms responsible for the upregulation of Bcl-2 upregulation, we investigated related signaling pathways and found that AKT phosphorylation was upregulated after Eya2 overexpression and was inhibited following the knockdown of Eya2. The PI3K/AKT pathway has been previously reported to induce Bcl-2 and inhibit apoptosis in human prostate cancers [22, 23]. To validate this, we used an AKT inhibitor to block its phosphorylation and found that the suppression of AKT phosphorylation abolished the effect of Eya2 upon Bcl-2. Consequently, our data supported the role of the Eya2/AKT/Bcl-2 axis in prostate cancer cells.

Collectively, our current data revealed that EYA2 promoted the progression of prostate cancer, potentially via the AKT/Bcl-2 axis. Our data therefore identified Eya2 as a potential therapeutic target.

## Figures and Tables

**Table 1:** Distribution of Eya2 status in prostate cancer according to clinicopathological characteristics.

Characteristics	Number of patients	Eya2 low expression	Eya2 high expression	P
Age				
<65	39	10	29	0.2288
≥65	59	22	37	
TNM stage				
I-II	53	24	29	0.0038
III-IV	45	8	37	
Gleason score				
≤7	29	14	15	0.0128
>7	66	15	51	
T stage				
T1-T2	69	28	35	0.0008
T3-T4	35	4	31	
Lymph node metastasis				
Absent	71	23	48	0.4965
Present	24	6	18	

## Data Availability

The data used to support the findings of this study are available from the corresponding author upon request.
